# Patterns of database citation in articles and patents indicate long-term scientific and industry value of biological data resources

**DOI:** 10.12688/f1000research.7911.1

**Published:** 2016-02-11

**Authors:** David Bousfield, Johanna McEntyre, Sameer Velankar, George Papadatos, Alex Bateman, Guy Cochrane, Jee-Hyub Kim, Florian Graef, Vid Vartak, Blaise Alako, Niklas Blomberg

**Affiliations:** 1 ELIXIR, Wellcome Genome Campus, Cambridge, UK; 2 Ganesha Associates, Cambridge, UK; 3 European Molecular Biology Laboratory, European Bioinformatics Institute (EMBL-EBI), Wellcome Genome Campus, Cambridge, UK

**Keywords:** Data citations, Data reuse, Data repositories, Data archiving, Open data, Bibliometrics, Patent analysis, Research impact

## Abstract

Data from open access biomolecular data resources, such as the European Nucleotide Archive and the Protein Data Bank are extensively reused within life science research for comparative studies, method development and to derive new scientific insights. Indicators that estimate the extent and utility of such secondary use of research data need to reflect this complex and highly variable data usage. By linking open access scientific literature, via Europe PubMedCentral, to the metadata in biological data resources we separate data citations associated with a deposition statement from citations that capture the subsequent, long-term, reuse of data in academia and industry.  We extend this analysis to begin to investigate citations of biomolecular resources in patent documents. We find citations in more than 8,000 patents from 2014, demonstrating substantial use and an important role for data resources in defining biological concepts in granted patents to both academic and industrial innovators. Combined together our results indicate that the citation patterns in biomedical literature and patents vary, not only due to citation practice but also according to the data resource cited. The results guard against the use of simple metrics such as citation counts and show that indicators of data use must not only take into account citations within the biomedical literature but also include reuse of data in industry and other parts of society by including patents and other scientific and technical documents such as guidelines, reports and grant applications.

## Introduction

Open sharing of data is a well-established norm in molecular biology and the genomic sciences: protein structure datasets are released to the community after the corresponding articles are published, many genome sequencing projects deposit sequences in public archives as soon as they are acquired. Consequently, the bioinformatics databases holding these data ^1^ form an essential part of molecular biology research. The standardisation, organisation and careful annotation that occurs when experimental data is deposited in openly accessible biomolecular resources such as the European Nucleotide Archive ^2^ or the Protein Data Bank ^3^ enables independent data verification and also support and encourage data reuse by the research community. The deposition of experimental data in structured archives is complemented by a long tradition of manual curation in which protein properties, biological reactions, genetic linkages and other facts from the scientific literature are further catalogued into structured reference collections such as UniProt ^4^, RefSeq ^5^, and OMIM ^6^. Value-adding data resources build on, and further combine, this treasure-trove of open data and provide comprehensive coverage of biology by cataloguing model organisms, protein classes, sequence motifs, biological pathways, reactions, metabolites: to date over 1600 biological databases are reported in the Nucleic Acids Research database catalogue ^7^.

Maintaining and updating an infrastructure to support the active collection, annotation and redistribution of data is costly and only makes sense if there is a research community that actively reuses the data. While the value of opening up data for independent validation is seen as imperative for the scientific debate ^8^, the open datasets from molecular biology research have long been used to stimulate and test additional hypotheses that are independent of the original experiment. The aggregation and inter-linking of published datasets also forms the basis for meta-analysis, modelling or new derivative databases ^9– 13^. Hence, managing these resources in an effective and sustainable manner requires database owners and funders to understand their usage and role in scientific research, as well as their role in generation of downstream societal value, for example by contributing to the definition of intellectual property held in patent documents. Quantitative analysis of data citation in scientific articles currently lacks metrics that parallel traditional scientific article citation indices and journal impact factors. Furthermore publishers of scientific journals rarely annotate database citations leaving organisations such as Europe PubMed Central (EPMC) to provide routine text-mining to find citations of database identifiers in full-text articles ^14^.

Estimating the on-going use of biological data resources by means of their citation patterns in scientific articles captures one aspect of data reuse but is challenging because data citations in the scientific literature are highly variable with few established community norms. For example, Piwowar and colleagues ^15^ tracked the citation practices used by three life science data resources: NCBI’s GEO ^16^, Pangaea ^17^, and Treebase ^18^. They manually curated data citation statements in a corpus of data-citing papers and noted that for datasets from Pangaea the norm was data citation via the reference list while for the other resources a significant proportion of the citations were made by direct mention of the unique data resource identifier in the text narrative. This variable citation practice, and the subsequent problem this poses for estimations of data usage by tracking data citations in the literature is further exemplified by Belter in a study of the data citation practices used by oceanographers ^19^. Despite the fact that the datasets studied had unambiguous terms of use, including recommendations for citation, the citation practices observed were highly variable with most citations occurring as a direct reference in the main text of the journal article. For example, Belter found that the editions of the National Oceanographic Data Center climate data set were cited in no less than 1180 different ways within his curated literature corpus.

Despite these challenges Kafkas *et al.* ^14^ have shown that text-mining database citation identifiers, i.e. the juxtaposition in the text of a database name and an appropriate accession ID, from an Open Access literature corpus within EPMC doubled the number of data citations compared to the number supplied by publishers. Subsequently they extended their study with an analysis of the supplementary material associated with the same corpus and noted that data citation practices in supplementary data files differed markedly from those observed in the main article ^20^. For instance, supplementary files often contain long lists of database identifiers. The rank of databases when ordered by the frequency of data citations also differed in supplementary data files compared to that observed in the main articles from the same corpus.

Collectively these studies give us a general sense of the scale of data use although the highly diverse citation practices observed cautions against a naive application of data citation as a metric for research impact. Furthermore, the statistics generated by the studies described above do not discriminate between citations arising from the initial deposition and publication of a source article and subsequent secondary citations in the research literature. Nor do these studies describe the flow and indirect use of data through the web of existing bioinformatics data resources. Thus there is a need to further investigate data citations to serve as a background for development of usage metrics, guide the life-cycle management of resources and understand the flow and impact of biological data. We build on and extend earlier studies by demonstrating how primary data citations, arising from the deposition of data and its citation in the source article describing the generation of the data, can be separated from subsequent secondary data citations. In this study we focussed our attention on two of the major biomolecular databases, the European Nucleotide Archive (ENA) and the Protein Data Bank (PDB), where the high-quality curation and well-established links between an open literature resource (EPMC) and data resources allow us to dissect primary from secondary data citations. We have done this by combining accession publication data from the biomolecular resources with the citation data from EPMC in order to provide an insight into dynamics of data citations over time. We further extend our study of data citations by mining a corpus of full-text patent documents (accessed via SureChEMBL ^21^) in order to begin to understand the downstream use of data resources in the definition of biological entities and concepts in a legal/technical commercial environment.

## Methods

### Sources of full-text and data accession citations used in this study

The full-text research articles used in this study were accessed from EPMC ^22^. The content scope of EPMC covers over 25 million PubMed abstracts and 3.5 million full-text articles (see https://europepmc.org/About), each article is identified by a unique identifier (a “PMID” for abstracts and a “PMCID” for full-text). Data accession references were extracted using EPMC’s text-mining pipeline based on a combination of rule-based knowledge about possible accession number structures and an empirically-determined set of contextual cues ^14, 20, 23^. The pipeline is integrated into the EPMC infrastructure ( http://europepmc.org/) and is used to identify instances of data citation in full-text articles on a daily basis. The data citations are made publically available via EPMC’s APIs. When comparing research articles with patents we focused on 2014 as the most recent year available. However, due to the fact that embargoed articles were still being added at the time of our study, we repeated our analysis using material from 2012 and 2013 to ensure that our comparisons were robust.

The Protein Data Bank (PDB) is the global archive of 3D structures of proteins, nucleic acids and complex assemblies. This large corpus of data (94,117 holdings in 2014) and related citations provide an extensive test set for developing and understanding data citation and access metrics ( http://www.wwpdb.org/stats/deposition). We used the European site PDBe as the definitive source of deposition data, i.e. accession identifiers, deposition dates and associated PMID publication details.

The European Nucleotide Archive (ENA; http://www.ebi.ac.uk/ena) is Europe's primary resource for nucleotide sequence information. The current size of the ENA is in excess of 2.5 petabytes, with a doubling time of approximately 20 months (see http://www.ebi.ac.uk/ena/about/statistics). We used ENA as an additional definitive source of deposition data, i.e. accession identifiers, deposition dates and associated PMID publication details.

SureChEMBL ( https://www.surechembl.org/) is a publicly available, large-scale resource containing chemical annotations found in the full-text, images and attachments of patent documents ^21^. Its data content at 28 October 2015 included more than 14 million chemically annotated full-text patent documents. In addition, it contains 130 million patent abstracts from DOCDB, the European Patent Office master documentation database with worldwide coverage containing bibliographic data, abstracts, and citations (but no full-text or images).

SureChEMBL provides full-text searching of the patent literature using a keyword-based querying functionality, complemented by a chemistry-based query engine. Our queries retrieved full-text patent documents (both applications and granted patents) written in the English language, published in 2014 by the three main patent authorities, namely the European Patent Office (EPO), the US Patent and Trademark Office (USPTO) and the World Intellectual Property Organisation (WIPO). To ensure the relevance of the retrieved patent documents to biological and life sciences, the appropriate international patent classification (IPC http://www.wipo.int/classifications/ipc/en/) codes (predominantly from categories A (human necessities) and C (chemistry), full query: “(ic:(A01 OR A23 OR A24 OR A61 OR A62B OR C05 OR C06 OR C07 OR C08 OR C09 OR C10 OR C11 OR C12 OR C13 OR C14 OR G01N) OR cpc:(A01 OR A23 OR A24 OR A61 OR A62B OR C05 OR C06 OR C07 OR C08 OR C09 OR C10 OR C11 OR C12 OR C13 OR C14 OR G01N) OR ecla_ec:(A01 OR A23 OR A24 OR A61 OR A62B OR C05 OR C06 OR C07 OR C08 OR C09 OR C10 OR C11 OR C12 OR C13 OR C14 OR G01N)) AND desc:the AND pdyear:[2010 TO 2014] AND pnlang:EN AND pnctry:(WO OR EP OR US)” ) were used to filter the results ^24^. No further selection was carried out on the basis of patent kind (an indication of where the patent is in the review process, e.g. application stage, or granted). Patent families were identified using the simple patent family definition provided by the European Patent Office (EPO) ^25^ and a single example selected at random to be sole representative of the group in subsequent analysis. In total, 188,589 documents published in 2014 were retrieved and used as input for the identifier extraction process. The XML content generated by these patent selections was then mined for accession numbers using the EPMC text-mining pipeline.

### Text-mining performance characteristics

The performance assessment characteristics of the text-mining pipeline have been previously reported as 97.45% precision/59.6% recall for ENA and 94.63% precision/91.36% recall for PDB accession references when calibrated against an open access full-text corpus from EPMC ^14^. No large-scale validation of the pipeline has been performed on the patent literature. However, manual inspection on a subset of 110 entries indicated that the approximate precision of the system was 99% and recall was 93%. Overall then the accuracy of the system appears to be higher when working with patents. This is possibly due to that fact that most citation-positive patents contain multiple exemplars whereas many research articles only include one. This would reduce the incidence of false negatives.

### Metadata acquisition

The EPMC metadata and text-mining results used in this study can be accessed or generated via Europe PMC’s RESTful API which gives access to search tools with citation-count sort order and data citation features. For example, to get all the PDB citations text-mined in the articles published in EPMC in 2014 go to http://www.ebi.ac.uk/europepmc/webservices/rest/search?query=PUB_YEAR:2014 and then for each of those get the accessions identifiers (e.g. for PMID 22517515 the query is http://www.ebi.ac.uk/europepmc/webservices/rest/MED/22517515/textMinedTerms/ACCESSION). The ENA accession data used here was obtained from EMBL release 124 (described in detail here ftp.ebi.ac.uk/pub/databases/ena/sequence/release/doc/relnotes.txt). The data are public and available at: ftp.ebi.ac.uk/pub/databases/embl/release/ or through the ENA Browser and REST API. We used the primary accession identifiers and deposition article PMIDs found in the flat XML files for each entry, and included all ENA data classes with the exception of the WGS (whole genome shotgun) depositions because these are lower level assemblies with sparse or no annotation information and so less likely to be cited in publications.

The PDB data was obtained from the 2 September 2015 release of the PDB. PDB has a weekly release cycle that is loaded and processed by the PDBe team. The PDBe database also contains information extracted from EPMC about additional PMID that reference or mention any given PDB accession identifier. This information is updated once a month. Citation data was extracted from the PDBe database and included information on PMID that mention the PDB identifier code or cite the primary citation describing the given PDB entry. Citation data is available via the PDBe API (See related publication call at http://www.ebi.ac.uk/pdbe/api/doc/) as well as on the individual PDBe entry pages (e.g. http://pdbe.org/3p8c and http://pdbe.org/3p8c/citations).

### Data analysis

Each record in a database has a unique accession number, a release or publication date, a series of revision dates, the bibliographic details of the deposition article, subsequent references associated with the generation of the data set and a list of references citing the source. By combining the metadata within the data resource with the citation information from EPMC we could identify the citation linked to the deposition article and hence distinguish between the initial citation event associated with the deposition article or the release of the data to the public, and track the secondary citations of a data entry (or annual cohorts of data entries) over time.

**Figure 1.**
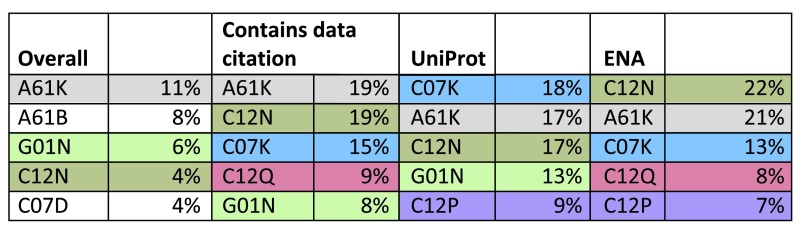
The prevalence of the top 5 four character IPC categories for the data set as a whole, those patents containing a data citation, and those patents having a data citation to UniProt or ENA. Note individual patents can have several IPC annotations – these percentages are based on summing all instances, i.e. “one code, one vote”. For example, 17% of the IPC codes annotating UniProt-positive patents were A61K. Key to coding: A61K preparations for medical, dental, or toilet purposes; C12N micro-organisms or enzymes; A61B diagnosis, surgery, intervention; C07K peptides; G01N investigating or analysing materials by determining their chemical or physical properties; C12P fermentation or enzyme-using processes to synthesise a desired chemical compound; C12Q measuring or testing processes involving enzymes or micro-organisms; C07D heterocyclic compounds. Note absence of A61B from the more biological data sets, compared to the presence of C07K.

The data sets associated with the generation of Figure 1, Table 1, Table 2, Table 3 and Table 4, and Supplementary material Table 1 and Supplementary material Table 2 are provided (see Data availability). More specifically, data sets containing accession identifiers, deposition_PMID, deposition year, year of first_publication, and publication year of PMID were extracted from the data resources, and corresponding accession identifiers, citation year and number of data citations in that year were extracted from EPMC.

The merged data set contained the variables: [accession_id], [deposition_pmid], [deposition_year], [first_public_year], [pmid_publication_year], [citation_year], [citations]. For records that had a [pmid_publication_year] equal to a [citation_year], we reduced the corresponding [citations] count by one to remove the impact of the deposition citation. We then tabulated total citations for [first_public_year] (or [pmid_publication_year]) against accession/source article [citation_year].

Our data analysis was carried out using the STATA 12 package ( http://www.stata.com/products/).

Raw data for ‘Patterns of database citation in articles and patents indicate long-term scientific and industry value of biological data resources’, Bousfield *et al.*, 2016 README.txt contains an index to the accompanying datasets: definition of the data fields is given along with a short STATA do routine. Click here for additional data file. Copyright: © 2016 Bousfield D et al. 2016 Data associated with the article are available under the terms of the Creative Commons Zero "No rights reserved" data waiver (CC0 1.0 Public domain dedication).

## Results

### Secondary citation of data from biomolecular resources

To establish a baseline, we used citations of accession identifiers captured by the EPMC text-mining pipeline to provide a comprehensive picture of the annual data citation characteristics for ENA ^26^, UniProt ^4^, PDBe ^3^, OMIM ^6^, RefSNP, RefSeq ^5^, Pfam ^27^, InterPro ^28^, Ensembl ^29^, and ArrayExpress ^30^.

In 2014, the ENA, PDB, and RefSNP accounted for 42.6%, 21.9% and 21.7% respectively of the total text-mined citations ( Table 1). These proportions remained approximately constant throughout the sampled periods and hence provide a reference for comparison with the patent corpus below. In the Kafkas *et al.* study ^14^ the corresponding percentages for a cohort of 486,472 articles published between 1990 and 2012 were 56.5%, 19.9% and 13.8%. We believe that the differences in these percentages can be attributed to the age structures of the two data corpuses, with the Kafkas set providing a more longitudinal view hence favouring well-established repositories such as ENA.

**Table 1.** Annual total accessions mined in Europe PMC full-text content published between 2012 and 2014, e.g. 7016 articles in 2014 contained 37,767 references to ENA accessions. Acc/Art is average accession references per article.

	Total accessions mined:			% Total	Articles:	Acc/
Repository	2012	2013	2014	2014	2014	Art
**ENA**	35897	33177	37767	42.6%	7016	5.4
**PDB**	21198	22047	19461	21.9%	5913	3.3
**RefSNP**	20528	21636	19252	21.7%	3638	5.3
**UniProt**	2308	2766	3925	4.4%	846	4.6
**OMIM**	1867	2051	2847	3.2%	819	3.5
**DOI**	445	914	1511	1.7%	1215	1.2
**RefSeq**	1148	1028	1484	1.7%	451	3.3
**Pfam**	896	1063	1190	1.3%	420	2.8
**ArrayExpress**	534	569	612	0.7%	419	1.5
**Ensembl**	204	293	389	0.4%	116	3.4
**Interpro**	139	224	269	0.3%	67	4.0
**Total**	85164	85768	88707	100%	20920	4.2

Unsurprisingly, given the breadth of the biomedical literature, data citations of individual biomolecular resources are relatively infrequent in EPMC: for ENA, the proportion of citing papers in 2014 are 7,016/319,815, or 2.2%, and for PDB, 5,913/319,815 or 1.8%. Collectively the investigated databases are referenced in 6.5% of our EPMC sample (the EPMC search: “pub_year:2014 in_epmc:y”, conducted 20 Oct 2015, retrieved 319,815 articles).

Estimates of secondary data citation in the scientific literature, whether measured via citation of an accession identifier in the article text or mentioned in the reference list (e.g. “1fho” or “doi: 0.2210/pdb1fho/pdb”) or via citation of the corresponding deposition article (e.g. “Blomberg *et al.* ^31^”), need to make a distinction between citations that arise from the original act of data deposition and those that arise from the secondary citation of data. A further distinction, not investigated systematically here, could also be made according to whether the article citations come from one or more of the original author group – as above - or from an independent research group. The former practise would appear to be quite common for ENA depositions (D. Bousfield, unpublished observations). While this distinction seems straightforward in principle, different policies and deposition practices, as well as ambiguity of author names, make it difficult to distinguish these alternatives in a large-scale analysis. We note that the adoption of ORCID within publication workflows will support future disambiguation.

Combining metadata stored in EPMC and the data resources allowed us to build up a picture, based on the summation of individual data elements, of how annual cohorts of accessions and deposition articles are cited over time (see Table 2). For example the PDB accession 2jhr that refers to the crystal structure of myosin-2 motor domain in complex with ADP-metavanadate and pentabromopseudilin was made public on 13 January 2009. The corresponding article for this deposition is PMID:19122661, entitled “The mechanism of pentabromopseudilin inhibition of myosin motor activity”, published later in 2009 ^32^. At the time of our study, the deposition article had been cited a total of 9 times during the period 2009–2014 (the current list of citing articles can be found in EPMC using the query: cites:19122661_med). None of these papers cite the data accession identifier 2jhr. However, the database record was cited once by its accession identifier in PMID:21841195 (PMCID:3186370), “Structural basis for the allosteric interference of myosin function by reactive thiol region mutations G680A and G680V”. The actual statement from this paper provides a good example of how data citation occurs in narrative text: *“This is very unusual, as the meta-vanadate is clearly visible in known wild-type myosin-2 structures that were obtained in the presence of ADP-VO3, like e.g. **PDB** IDs 2JJ9, **2JHR**, and 2XO8”* ^33^. The text components recognised by the text-mining pipeline as being an accession citation of 2JHR are highlighted in bold. The text-mining pipeline also found 2JJ9 and 2XO8.

**Table 2.** PDB accession citations by annual publication cohort. The rows show the year in which a PDB data entry was first made public. The columns denote the year in which a citation of that data accession was recorded. Thus each row displays the time-series of citations for the cohort of data entries published during a given year. Reasons why there are observations below the diagonal are discussed in the text. Mature cohorts (release years 2005–2011) were cited on average 0.21 times per accession per year.

PDB RELEASE YEAR	NEW ACCESSIONS PUBLISHED		SUBSEQUENT CITATION OF ACCESSION IN EPMC								
		2005	2006	2007	2008	2009	2010	2011	2012	2013	2014
**2005**	4,165	79	396	675	1,093	1,189	1,243	1,273	1,362	1,199	1,036
**2006**	4,930	6	93	485	998	1,211	1,290	1,274	1,253	1,300	1,074
**2007**	5,113	0	6	141	768	1,181	1,292	1,309	1,302	1,337	1,208
**2008**	5,255	2	1	14	179	998	1,308	1,335	1,468	1,308	1,320
**2009**	5,478	0	1	0	6	206	1,024	1,355	1,408	1,402	1,270
**2010**	5,792	1	1	0	2	8	254	1,123	1,495	1,483	1,432
**2011**	5,854	0	1	1	4	3	5	284	1,055	1,420	1,294
**2012**	6,309	1	2	2	0	1	4	12	331	1,232	1,488
**2013**	5,798	0	2	2	1	5	3	0	6	339	1,145
**2014**	2,152	0	0	0	0	1	0	0	0	3	176

Note that whereas each data resource by definition contains references to the complete set of deposition articles, EPMC is incomplete in its full-text literature coverage and therefore will contain only a partial set of cited accession identifiers. In addition, the text-mining process will miss some citations (false negatives) and potentially create a small number of false positives (see Methods). These factors need to be kept in mind when interpreting the results shown in Table 2 and Table 3.

**Table 3.** PDB source article citations by annual publication cohort. Same format as per Table 3. Notice sustained levels of citation over time. Mature cohorts (publication year 2005–2011) were cited on average 6.73 times per source article per year.

SOURCE ARTICLE PUBLICATION YEAR	NEW SOURCE ARTICLES		SUBSEQUENT CITATION OF SOURCE REFERENCE IN EPMC								
		2005	2006	2007	2008	2009	2010	2011	2012	2013	2014
**2005**	2,232	3,033	11,275	14,382	15,918	15,891	16,548	16,068	14,830	16,290	12,019
**2006**	2,421	10	3,233	13,330	16,464	17,489	17,422	17,163	15,939	17,293	12,635
**2007**	2,566	6	2	4,066	17,634	21,476	22,020	22,555	19,965	22,003	15,839
**2008**	2,596	9	14	49	3,840	17,567	21,813	21,366	20,129	21,749	16,014
**2009**	2,645	3	0	3	10	4,813	19,942	23,826	22,667	24,648	17,583
**2010**	2,787	3	0	2	7	0	5,204	22,206	25,781	27,645	20,553
**2011**	2,782	0	0	1	2	0	4	5,441	23,172	30,283	23,358
**2012**	2,895	0	0	0	0	0	0	9	6,951	32,529	28,939
**2013**	2,665	6	0	0	0	2	2	5	3	8,408	26,067
**2014**	957	1	0	0	0	0	0	0	5	39	4,659

Table 2 displays the secondary citation of PDB accession identifiers, subject to above-mentioned caveats, published between 2005 and 2014. In theory, elements below the diagonal should all be zero as the non-zero numbers imply that the accession identifier has been cited before it has been made public. Some of these “below the diagonal” observations may be true false positives created by the text-mining process but also occur when the primary reference article in the database has been updated to a more recent publication. Non-zero “below diagonal” citations can also arise when authors embargo the publication of the data until after the publication of their own additional work citing the data set.

Table 3 shows the corresponding picture for the continuing citation of the PDB deposition articles. Some similar “below diagonal” patterns were found and attributed to use of updated primary reference articles or occasionally genuine misalignments of the underlying archives.

As can be seen from Table 2 and Table 3 the citation of PDB data accession identifiers and PDB deposition articles remain high as the annual cohorts age. The average annual citation-rate for each deposition article in PDB is 6.7 and the annual average number of citations per accession identifier is 0.2. For ENA the corresponding statistics were 2.1 and 0.1 ( Supplementary material Table 1 and Supplementary material Table 2 show the corresponding two data sets for ENA). In all four cases these citation rates are stable over time. It is worth noting that most ENA data depositions are not accompanied by a deposition article: 32,188,662 ENA entries in 2014 were not associated with a deposition article as compared to the 26,384,613 entries that were associated with 9,375 source articles. It is also worth noting that the text-mining of accession numbers in EPMC only occurs in the subset of the scientific literature where full-text is available in open access resources, hence these numbers represent a lower bound on direct data citations.

### Biological data resources are extensively used within patents

Patents are frequently used as an indicator of broader societal value of research ^34– 37^. Importantly, it is estimated that only a small fraction of the scientific and technological innovation first reported in patents is subsequently disclosed in scientific literature sources ^38^. During the creation of a patent it is essential to unambiguously identify the components of the invention and to provide extensive reviews of any prior art ^39^. Thus we sought to address the question of how these requirements translate into data citation practices within patents.

Our SureCHEMBL corpus of 188,589 full-text patents contained 7,923 patents with data citations (4.2% of the corpus). Data citations were most common in the description section – which usually constitutes by far the largest section of the document text. The breakdown by patent office shows that the majority of patents with data citations were from the US (see Supplementary material Table 3). The proportion of accessions found for the different repositories ( Table 4) differed considerably from that of EPMC articles (see Table 1 for comparison) with RefSeq, ENA, RefSNP and UniProt dominating. The average number of cited accession identifiers per repository and per document (13.9) and the variance of these figures across the resources was also much higher than found for the full-text scientific literature corpus. Since the international patent code (IPC, see Methods) is a hierarchical patent classification system we can use its additional levels to probe the subject matter of accession-positive patents further. Figure 1 shows that patents with references to ENA and UniProt were extensively used to define biological entities in the IPC subclasses A61 (“Preparations for medical, dental or toilet purposes”), C07 (“Organic chemistry”) and C12 (“Microorganisms or enzymes”). The content profiles and scientific topics covered in the two corpuses – open access scientific publications and patent documents - are different and further work is needed to understand how this influences data citation rates.

**Table 4.** Data citations mined from a 2014 SureCHEMBL patent cohort. Compare the averages with those in Table 1. Acc/Pat is the average number of accessions per patent per repository.

Repository	Accessions	%Total	Patents	Acc/Pat
**RefSeq**	34,634	30%	1,002	34.6
**ENA**	33,097	28%	4,074	8.1
**RefSNP**	26,206	22%	322	81.4
**UniProt**	14,127	12%	1,387	10.2
**PDB**	3,612	3%	1,093	3.3
**Ensembl**	1,877	2%	97	19.4
**OMIM**	1,769	2%	254	7.0
**Pfam**	1,158	1%	115	10.1
**Interpro**	601	1%	46	13.1
**ArrayExpress**	30	0%	19	1.6
**Total**	117,111		8,409	19

## Discussion

Citation analysis is a cornerstone of research impact and evaluation and while the use and value of citation of research papers in the scholarly literature as a metric for research is much debated, the citation practices underpinning such analysis are generally unambiguous and well established. With research funders increasingly establishing open data policies, there is a requirement and interest in performance metrics that assess the reuse of open research data - whether to recognise and reward scientists, support the long-term management and sustainability of data archives or to understand the broader societal value derived from these policies. Quantitative analysis of data reuse, let alone estimating the value arising from this reuse, is challenging due to the diversity of data citation practices but also due to the many ways open research data can be used in further studies. As bioinformatics databases increasingly take on the role of dictionaries or “scientific instruments” ^40^ we would expect that most of the use of biomolecular data resources (and consequently data reuse from these resources) is never cited, just as most literature searches, views or downloads from PubMed do not lead to a citation of the PubMed infrastructure.

This study set out to analyse data citation practices with the aim of describing secondary citation of data entries – as one indicator of data reuse - in full-text content available from EPMC (scientific papers) and SureCHEMBL (patents). We focused our efforts on the major biomolecular databases where high-quality curation processes and well-established links between literature and data resources allows us to dissect citations arising from data deposition articles from the secondary citations arising from reuse of this dataset in the scientific literature. Our approach can in principle be applied to all repositories by systematically bringing together metadata from the repository and from EPMC and is in itself a good illustration of the value that open access data and literature resources brings to the scientific community.

The need to separate deposition from reuse in quantitative studies of data citation has been noted previously ^41^ but the complexity and manual analysis required often leads investigators towards aggregate analysis of a total citation rate. For instance, in an analysis of data citation practices across fields using the commercially available Thomson-Reuters Data Citation Index ^42^ the average citation rate for data sets in many of the studied data resources was found to be close to one, suggesting that much of the ‘data citation’ found in this analysis was driven by data deposition publications. Separating out secondary citations by tracking them over time ( Table 2 and Table 3) provides one, albeit limited, indicator of the reuse of the data sets in the scientific community. In the case of the two repositories we have analysed in detail, PDB and ENA, it demonstrates long-term reuse of data sets by the community.

Comparing the citation patterns arising from the deposition and reuse from ENA and PDB is instructive, as the mode of usage is very different for the two databases. While ENA is accessed directly by users on a daily basis, the more significant use is as a large reference repository that serves as the archival backend for user-focussed resources such as the genome browsers Ensembl and Ensembl genomes ^29^. Most of the users that access the Ensembl resource on a daily basis are likely to be unaware of the relationship between ENA and Ensembl and hence would not cite the corresponding ENA entry.

The results in this study, taken together with previous work ^15, 19, 40, 43^ guards against reliance on metrics based on familiar approaches developed for the analysis of scientific papers. Such simplified citation metrics do not capture the many different forms of data reuse and heterogeneous and non-standard data citation practice in the biomedical literature. Data citation indexes also need to be developed that acknowledge that different patterns of use give different citation patterns for archival resources (e.g. PDB, ENA, GEO), reference knowledge bases (e.g. UniProt, Reactome, Human Protein Atlas), and secondary value-added resources (e.g. Interpro). Uniform quantitative indicators of data citation are inappropriate as they do not capture the usage patterns of the different resources.

Biomolecular databases also exist within a network of mutual referencing and cross-mappings - just as literature articles build upon previous scholarly work and indicate this through citation there is a complex network of dependencies between bioinformatics databases - most of which is not visible in the primary literature ^44^. Further work is needed to capture this usage pattern for assessments of the data journeys that occur through the extensive reuse and cross referencing of bioinformatics resources – and the corresponding return of investment from this scientific infrastructure.

To date investigations on data reuse have focused on the scientific literature. However, biological data resources are also extensively used by researchers in industry and in the second part of our study we started to address the use of bioinformatics databases in patents as a broader indicator of their industrial and societal value. Patent analyses have been extensively used to understand the industry and societal benefit from publicly funded research ^37, 45– 47^ and full-text patents are available from several patent offices. The practice of large-scale text-mining of molecular entities from patents is well-established in chemistry ^48– 51^. However, to the best of our knowledge this is the first time that the usage and citation of bioinformatics data resources in the patent literature has been analysed; our beginning foray into this field demonstrates significant use of these resources to define biological concepts and subject matter in patent documents. Although the majority of data citation occurs in patent classes dealing with pharmaceutical and medical inventions (drugs, diagnostics and medical devices) the data also highlights a broad applicability of biomolecular resources in bio-based industries with usage in industrial biotechnology and consumer products, for example the definitions of enzymatic activity in washing powder.

## Conclusion

The extensive and quantifiable reuse of data from biomolecular data resources demonstrates the critical role this infrastructure plays in life science research but also highlights the need for robust metrics of data use by the scientific community. Using the cross-referencing between literature repositories such as Europe PMC and the ENA and PDB archives we demonstrate how data citations arising from deposition of data in an archive can be distinguished from the subsequent reuse by the scientific community – an important distinction in research evaluation as the former provides an estimate of adoption of community best practice and/or compliance with open access guidelines, whereas the second is an indicator of the value created by these practices and guidelines. The study also demonstrates that measures based on literature citation may be more or less informative according to mode of use of a repository: large biological archives serve as foundations for other value-added resources. Individual data items from large repositories such as ENA may not be directly cited in the scientific literature but collectively forms important reference collections for e.g. pathogen detection or biodiversity research. Further work is needed to develop methods that classify and account for this mode of use, e.g. by quantifying database cross-linking via literature citation networks and identifier mapping.

By extending the analysis to patent documents we show that the biological data resources provide unambiguous definitions of biological entities for use in official documents such as patents. This shows that life science data resources transcend basic research and form a fundamental component of the digital knowledge management framework needed in a modern society. Hence, assessment of the use and value of scientific data repositories should include data from research articles, patents and perhaps other documents of record such as clinical guidelines, standards, and grant applications. Understanding how to establish robust indicators of data citation in these types of documents in addition to research articles remains an important challenge for further studies. The ecosystem of open literature and data resources can only be sustained if the creation of scientific and societal value can be properly assessed and the scientific and scholarly community needs to make a concerted effort to better cite data. Similar principles can be applied to other resources such as reagents and software. Finally we note that the insights from reviewing data citation patterns could be used to improve article level metrics, this is also an area of further investigation.

## Data availability

The data referenced by this article are under copyright with the following copyright statement: Copyright: © 2016 Bousfield D et al.

Data associated with the article are available under the terms of the Creative Commons Zero "No rights reserved" data waiver (CC0 1.0 Public domain dedication). 

*F1000Research:* Dataset 1. Raw data for ‘Patterns of database citation in articles and patents indicate long-term scientific and industry value of biological data resources’, Bousfield *et al.*, 2016. 10.5256/f1000research.7911.d113281 ^52^

## Supplementary Material

Supplementary tables for 'Patterns of database citation in articles and patents indicate long-term scientific and industry value of biological data resources'.

## References

[1] Baxevanis AD Bateman A: The Importance of Biological Databases in Biological Discovery. *Curr Protoc Bioinformatics.* 2015;50:1.1.1–1.8. 10.1002/0471250953.bi0101s50 26094768

[2] Nakamura Y Cochrane G Karsch-Mizrachi I: The International Nucleotide Sequence Database Collaboration. *Nucleic Acids Res.* 2013;41(Database issue):D21–D24. 10.1093/nar/gks1084 23180798 PMC3531182

[3] Gutmanas A Alhroub Y Battle GM: PDBe: Protein Data Bank in Europe. *Nucleic Acids Res.* 2014;42(Database issue):D285–D291. 10.1093/nar/gkt1180 24288376 PMC3965016

[4] UniProt Consortium: UniProt: a hub for protein information. *Nucleic Acids Res.* 2015;43(Database issue):D204–D212. 10.1093/nar/gku989 25348405 PMC4384041

[5] Pruitt KD Brown GR Hiatt SM: RefSeq: an update on mammalian reference sequences. *Nucleic Acids Res.* 2014;42(Database issue):D756–D763. 10.1093/nar/gkt1114 24259432 PMC3965018

[6] Online Mendelian Inheritance in Man, OMIM ^®^ .2016 Reference Source 17642958

[7] Nucleic Acids Research Database Summary.2015 Reference Source

[8] Boulton R: Science as an open enterprise. London: Royal Society.2012;104 Reference Source

[9] Rebholz-Schuhmann D Grabmüller C Kavaliauskas S: A case study: semantic integration of gene-disease associations for type 2 diabetes mellitus from literature and biomedical data resources. *Drug Discov Today.* 2014;19(7):882–9. 10.1016/j.drudis.2013.10.024 24201223

[10] Schurer SC Vempati U Smith R: BioAssay ontology annotations facilitate cross-analysis of diverse high-throughput screening data sets. *J Biomol Screen.* 2011;16(4):415–426. 10.1177/1087057111400191 21471461 PMC3167204

[11] Gaulton A Overington JP: Role of open chemical data in aiding drug discovery and design. *Future Med Chem.* 2010;2(6):903–907. 10.4155/fmc.10.191 21426107

[12] Schwede T: Protein modeling: what happened to the “protein structure gap”? *Structure.* 2013;21(9):1531–1540. 10.1016/j.str.2013.08.007 24010712 PMC3816506

[13] Rung J Brazma A: Reuse of public genome-wide gene expression data. *Nat Rev Genet.* 2013;14(2):89–99. 10.1038/nrg3394 23269463

[14] Kafkas Ş Kim JH McEntyre JR: Database citation in full text biomedical articles. *PLoS One.* 2013;8(5):e63184. 10.1371/journal.pone.0063184 23734176 PMC3667078

[15] Piwowar HA Carlson JD Vision TJ: Beginning to track 1000 datasets from public repositories into the published literature. *Proc Am Soc Info Sci Tech.* 2011;48(1):1–4. 10.1002/meet.2011.14504801337

[16] Barrett T Wilhite SE Ledoux P: NCBI GEO: archive for functional genomics data sets--update. *Nucleic Acids Res.* 2013;41(Database issue):D991–D995. 10.1093/nar/gks1193 23193258 PMC3531084

[17] PANGAEA: Data Publisher for Earth & Environmental Science. 10.1594/PANGAEA PMC10238520 37268655

[18] Sanderson MJ Donoghue MJ Piel WH: TreeBASE: a prototype database of phylogenetic analyses and an interactive tool for browsing the phylogeny of life. *Am J Bot.* 1994;81(6):183 Reference Source

[19] Belter CW: Measuring the value of research data: a citation analysis of oceanographic data sets. Browman HI, ed. *PLoS One.* 2014;9(3):e92590. 10.1371/journal.pone.0092590 24671177 PMC3966791

[20] Kafkas Ş Kim JH Pi X: Database citation in supplementary data linked to Europe PubMed Central full text biomedical articles. *J Biomed Semantics.* 2015;6(1):1. 10.1186/2041-1480-6-1 25789152 PMC4363206

[21] Papadatos G Davies M Dedman N: SureChEMBL: a large-scale, chemically annotated patent document database. *Nucleic Acids Res.* 2016;44(D1):D1220–D1228. 10.1093/nar/gkv1253 26582922 PMC4702887

[22] Europe PMC Consortium: Europe PMC: a full-text literature database for the life sciences and platform for innovation. *Nucleic Acids Res.* 2015;43(Database issue):D1042–D1048. 10.1093/nar/gku1061 25378340 PMC4383902

[23] Rebholz-Schuhmann D Arregui M Gaudan S: Text processing through Web services: calling Whatizit. *Bioinformatics.* 2008;24(2):296–298. 10.1093/bioinformatics/btm557 18006544

[24] Eisinger D Tsatsaronis G Bundschus M: Automated Patent Categorization and Guided Patent Search using IPC as Inspired by MeSH and PubMed. *J Biomed Semantics.* 2013;4(Suppl 1):S3. 10.1186/2041-1480-4-S1-S3 23734562 PMC3632996

[25] The Espacenet patent family.2015 Reference Source

[26] Silvester N Alako B Amid C: Content discovery and retrieval services at the European Nucleotide Archive. *Nucleic Acids Res.* 2015;43(Database issue):D23–D29. 10.1093/nar/gku1129 25404130 PMC4383942

[27] Finn RD Bateman A Clements J: Pfam: the protein families database. *Nucleic Acids Res.* 2014;42(Database issue):D222–D230. 10.1093/nar/gkt1223 24288371 PMC3965110

[28] Hunter S Jones P Mitchell A: InterPro in 2011: new developments in the family and domain prediction database. *Nucleic Acids Res.* 2012;40(Database issue):D306–D312. 10.1093/nar/gkr948 22096229 PMC3245097

[29] Flicek P Amode MR Barrell D: Ensembl 2014. *Nucleic Acids Res.* 2014;42(Database issue):D749–D755. 10.1093/nar/gkt1196 24316576 PMC3964975

[30] Petryszak R Burdett T Fiorelli B: Expression Atlas update--a database of gene and transcript expression from microarray- and sequencing-based functional genomics experiments. *Nucleic Acids Res.* 2013;42(Database issue):D926–D932. 10.1093/nar/gkt1270 24304889 PMC3964963

[31] Blomberg N Baraldi E Sattler M: Structure of a PH domain from the *C. elegans* muscle protein UNC-89 suggests a novel function. *Structure.* 2000;8(10):1079–1087. 10.1016/S0969-2126(00)00509-8 11080629

[32] Fedorov R Böhl M Tsiavaliaris G: The mechanism of pentabromopseudilin inhibition of myosin motor activity. *Nat Struct Mol Biol.* 2009;16(1):80–88. 10.1038/nsmb.1542 19122661

[33] Preller M Bauer S Adamek N: Structural basis for the allosteric interference of myosin function by reactive thiol region mutations G680A and G680V. *J Biol Chem.* 2011;286(40):35051–35060. 10.1074/jbc.M111.265298 21841195 PMC3186370

[34] Bessen J: The value of U.S. patents by owner and patent characteristics. *Res Policy.* 2008;37(5):932–945. 10.1016/j.respol.2008.02.005

[35] Narin F Hamilton KS Olivastro D: The increasing linkage between U.S. technology and public science. *Res Policy.* 1997;26(3):317–330. 10.1016/S0048-7333(97)00013-9

[36] Minguillo D Thelwall M: Which are the best innovation support infrastructures for universities? Evidence from R&D output and commercial activities. *Scientometrics.* 2015;102(1):1057–1081. 10.1007/s11192-014-1458-5

[37] Benson CL Magee CL: Quantitative determination of technological improvement from patent data. Huy NT, ed. *PLoS One.* 2015;10(4):e0121635. 10.1371/journal.pone.0121635 25874447 PMC4398537

[38] Bregonje M: Patents: A unique source for scientific technical information in chemistry related industry? *World Patent Information.* 2005;27(4):309–315. 10.1016/j.wpi.2005.05.003

[39] Grubb PW Thomsen PR: Patents for Chemicals, Pharmaceuticals, and Biotechnology. Oxford University Press;2010 Reference Source

[40] Hine C: Databases as Scientific Instruments and Their Role in the Ordering of Scientific Work. *Soc Stud Sci.* 2006;36(2):269–298. 10.1177/0306312706054047

[41] Piwowar HA: Foundational Studies for Measuring the Impact, Prevalence, and Patterns of Publicly Sharing Biomedical Research Data.2010 Reference Source

[42] Robinson-García N Jiménez-Contreras E Torres-Salinas D: Analyzing data citation practices using the Data Citation Index. *arXiv.*cs.DL:n/a-n/a.2015 10.1002/asi.23529

[43] Vision TJ Piwowar HA: Data reuse and scholarly reward: understanding practice and building infrastructure.2013 10.7287/peerj.preprints.14v1

[44] Cook CE Bergman MT Finn RD: The European Bioinformatics Institute in 2016: Data growth and integration. *Nucleic Acids Res.* 2016;44(D1):D20–D26. 10.1093/nar/gkv1352 26673705 PMC4702932

[45] Schankerman M Pakes A: Estimates of the Value of Patent Rights in European Countries During the Post-1950 Period. Cambridge, MA: National Bureau of Economic Research;1985 10.3386/w1650

[46] Bacchiocchi E Montobbio F: Knowledge diffusion from university and public research. A comparison between US, Japan and Europe using patent citations. *J Technol Transf.* 2009;34(2):169–181. 10.1007/s10961-007-9070-y

[47] Packalen M Bhattacharya J: Words in Patents: Research Inputs and the Value of Innovativeness in Invention. Cambridge, MA: National Bureau of Economic Research;2012 10.3386/w18494

[48] Jessop DM Adams SE Murray-Rust P: Mining chemical information from open patents. *J Cheminform.* 2011;3(1):40. 10.1186/1758-2946-3-40 21999425 PMC3205044

[49] Akhondi SA Klenner AG Tyrchan C: Annotated chemical patent corpus: a gold standard for text mining. *PLoS One.* 2014;9(9):e107477. 10.1371/journal.pone.0107477 25268232 PMC4182036

[50] Hersey A Chambers J Bellis L: Chemical databases: curation or integration by user-defined equivalence? *Drug Discov Today Technol.* 2015;14:17–24. 10.1016/j.ddtec.2015.01.005 26194583 PMC6294287

[51] Senger S Bartek L Papadatos G: Managing expectations: assessment of chemistry databases generated by automated extraction of chemical structures from patents. *J Cheminform.* 2015;7(1):49. 10.1186/s13321-015-0097-z 26457120 PMC4594083

[52] Bousfield D McEntyre J: Dataset 1 in: Patterns of database citation in articles and patents indicate long-term scientific and industry value of biological data resources. *F1000Research.* 2016 Data Source 10.12688/f1000research.7911.1 PMC4821287 27092246

