# Supplementary material for: Patterns of database citation in articles and patents indicate long-term scientific and industry value of biological data resources
Source: F1000Res. 2016 Feb 11;5:ELIXIR-160. [Version 1] doi: 10.12688/f1000research.7911.1 (PMC4821287; doi:10.12688/f1000research.7911.1)
Supplement: Supplementary file 2 [file f1000research-5-8516-s0001.tgz › c5cf34a2-33a7-45b9-846e-8338933d0cb7.docx]

**Supplementary material**

| **Table 1.** | | | | |  |  |  |  |  |  |  |
| --- | --- | --- | --- | --- | --- | --- | --- | --- | --- | --- | --- |
|  |  |  |  |  |  |  |  |  |  |  |  |
| ENA FIRST PUBLIC YEAR | NEW ACCESSIONS PUBLISHED | SUBSEQUENT CITATION OF ACCESSION IN EPMC | | | | | |  |  |  |  |
|  |  |  |  |  |  |  |  |  |  |  |  |
|  |  | **2005** | **2006** | **2007** | **2008** | **2009** | **2010** | **2011** | **2012** | **2013** | **2014** |
|  |  |  |  |  |  |  |  |  |  |  |  |
| **2005** | 13,339 | 85 | 628 | 803 | 973 | 733 | 764 | 579 | 556 | 381 | 470 |
| **2006** | 13,001 | 7 | 124 | 697 | 945 | 865 | 736 | 722 | 639 | 489 | 455 |
| **2007** | 14,825 | 1 | 0 | 195 | 698 | 1,149 | 688 | 902 | 495 | 551 | 674 |
| **2008** | 14,485 | 0 | 1 | 5 | 202 | 765 | 904 | 1,082 | 728 | 580 | 612 |
| **2009** | 13,297 | 3 | 3 | 3 | 13 | 179 | 874 | 1,073 | 914 | 820 | 708 |
| **2010** | 13,999 | 0 | 0 | 0 | 4 | 3 | 173 | 768 | 1,043 | 769 | 661 |
| **2011** | 14,753 | 0 | 0 | 0 | 0 | 0 | 19 | 241 | 777 | 923 | 930 |
| **2012** | 14,909 | 0 | 0 | 0 | 0 | 0 | 0 | 10 | 236 | 905 | 1,500 |
| **2013** | 11,755 | 0 | 0 | 0 | 0 | 0 | 0 | 6 | 5 | 366 | 878 |
| **2014** | 11,746 | 0 | 0 | 0 | 0 | 0 | 0 | 0 | 1 | 10 | 393 |

**Table 1.** Citation of ENA accession cohorts. The rows show the year in which a ENA data entry was first made public. The columns denote the year in which a citation was recorded. Thus each row displays the time-series of citations for the cohort of data

entries published during a given year. Reasons why there are observations below the diagonal are discussed in the text. ENA accessions in the mature cohorts (2005-2010) were cited on average 0.05 times per accession per year.

| **Table 2.** | | | | |  |  |  |  |  |  |  |
| --- | --- | --- | --- | --- | --- | --- | --- | --- | --- | --- | --- |
|  |  |  |  |  |  |  |  |  |  |  |  |
| ENA SOURCE PUBLICATION YEAR | NEW SOURCE ARTICLES PUBLISHED |  | SUBSEQUENT CITATION OF SOURCE REFERENCE IN EPMC | | | | | |  |  |  |
|  |  | **2005** | **2006** | **2007** | **2008** | **2009** | **2010** | **2011** | **2012** | **2013** | **2014** |
|  |  |  |  |  |  |  |  |  |  |  |  |
| **2005** | 10,413 | 4,887 | 21,706 | 27,047 | 28,338 | 27,461 | 27,721 | 28,273 | 26,778 | 30,114 | 24,534 |
| **2006** | 10,711 | 4 | 4,615 | 21,018 | 27,797 | 27,793 | 28,537 | 29,159 | 27,276 | 31,057 | 25,092 |
| **2007** | 10,237 | 0 | 5 | 4,096 | 20,976 | 26,094 | 27,873 | 28,759 | 27,004 | 30,791 | 25,206 |
| **2008** | 10,331 | 2 | 0 | 5 | 4,703 | 20,755 | 27,309 | 29,086 | 27,821 | 31,424 | 25,853 |
| **2009** | 11,167 | 0 | 0 | 0 | 5 | 5,572 | 23,678 | 30,792 | 29,416 | 33,803 | 27,764 |
| **2010** | 10,586 | 0 | 0 | 0 | 2 | 6 | 5,051 | 22,230 | 27,873 | 32,997 | 27,230 |
| **2011** | 10,744 | 0 | 0 | 0 | 3 | 1 | 7 | 5,368 | 24,267 | 35,175 | 29,804 |
| **2012** | 10,484 | 0 | 0 | 0 | 0 | 0 | 0 | 5 | 5,930 | 29,214 | 30,142 |
| **2013** | 7,983 | 0 | 0 | 0 | 0 | 0 | 0 | 0 | 7 | 6,427 | 22,436 |
| **2014** | 2,199 | 0 | 0 | 0 | 0 | 0 | 0 | 0 | 0 | 2 | 4,201 |

**Table 2:** ENA data citations by annual publication cohort. Mature cohorts (publication year 2005-2011) were cited on average 2.29 times per source article per year.

| **Table 3.** | |  |  |  |
| --- | --- | --- | --- | --- |
|  | **Patent Office** | |  |  |
| **Repository** | **EP** | **US** | **WO** | **Total** |
|  |  |  |  |  |
| **refseq** | 4,314 | 28,621 | 1,699 | 34,634 |
| **ena** | 4,154 | 26,200 | 2,743 | 33,097 |
| **refsnp** | 6,389 | 18,732 | 1,085 | 26,206 |
| **uniprot** | 1,500 | 10,830 | 1,797 | 14,127 |
| **pdb** | 368 | 2,651 | 593 | 3,612 |
| **ensembl** | 228 | 1,447 | 202 | 1,877 |
| **omim** | 210 | 1,372 | 187 | 1,769 |
| **pfam** | 409 | 724 | 25 | 1,158 |
| **interpro** | 415 | 177 | 9 | 601 |
| **arrayexpress** | 6 | 9 | 15 | 30 |
| **doi** | 2 | 0 | 2 | 4 |
|  |  |  |  |  |
| **Total** | 17,995 | 90,763 | 8,357 | 117,115 |

**Table 3.** Summary geo-statistics for repository citation from the 2014 SureChEMBL corpus. shown in Table 4. Breakdown by repository source and patent office. Key: EP = European Patent Office; US = US Patent Office; WO = World Patent Office.
