# Supplementary material for: Patterns of database citation in articles and patents indicate long-term scientific and industry value of biological data resources
Source: F1000Res. 2016 Feb 11;5:ELIXIR-160. [Version 1] doi: 10.12688/f1000research.7911.1 (PMC4821287; doi:10.12688/f1000research.7911.1)
Supplement: Peer review history

# Peer review: Referee response for version 1

**DOI:** 10.5256/f1000research.8516.r12982

**Published:** 2016-04-13

**Authors:** Ben Johnson

**Affiliations:** 1 Higher Education Funding Council for England (HEFCE), Avon, UK

Those involved in thinking about the scientific and societal impact of research will know that the complexities in data sharing, citation and reuse practices often hinder us from developing a quantitative understanding of the value of data. This has implications for how data are treated as an important output of research across a wide variety of fields, including in institutional and funder assessment frameworks.

The line of inquiry taken in this study to separate original citation from later reuse provides us with a one very promising way forward for how we might start to address some of these complexities. In this way, the study makes a valuable contribution to our understanding of how the impact of scientific data may usefully be quantified and tracked in future.

In addition, the study shows us the importance of open and carefully-maintained data infrastructure -- not just for data but also for scientific literature and patents - if we are to understand the important role that data plays in a modern, knowledge-based economy. The importance of high quality data sources is clear; the scope for others to extend this methodology to other disciplines and fields may depend on similar high quality sources and infrastructure being in place. If further work is needed to improve the data infrastructure then this study in part provides a good rationale for this.

This is a very well-designed study with a clear methodology. The paper is very well-written and the discussion section in particular provides a valuable and thought-provoking contribution to the debates.

I have read this submission. I believe that I have an appropriate level of expertise to confirm that it is of an acceptable scientific standard.

# Peer review: Referee response for version 1

**DOI:** 10.5256/f1000research.8516.r12390

**Published:** 2016-03-31

**Authors:** Timothy W. Clark

**Affiliations:** 1 MassGeneral Institute for Neurodegenerative Disease (MIND), Massachusetts General Hospital, Boston, MA, USA

This article is an important look at citation patterns and frequencies of some important and representative bioinformatics data resources (ENA, ePDB) in the professional and patent literature, based on textmining for accesssion numbers in the Euro PubMed Central Open Access corpus, and patent documents in SureChEMBL.

The authors provide resources to reproduce their results using RESTFUL APIs at EMBL.  

As might be expected of resource providers interested to know how the services they provide are taken up - the authors deal exclusively with “citation for reuse”.  By this is meant citation of database entry accession numbers to indicate they were used as a source of further analyses, rather than to mark them as an original primary data deposition. Analysis of citations for reuse documents the value of archived data for use beyond the study that produced it.

The methodology is well described. Authors note that “citation analysis is a cornerstone of research impact and evaluation”. Their analysis demonstrates distinct usage patterns for deposition and reuse in ENA and ePDB and for patents vs. articles as citing documents.

By quantifying the extent of reuse of data from EMBL bimolecular resources the authors demonstrate the importance in biomedical research of such resources .

This is a well written paper with a carefully designed methodology which quantifies the role of key EMBL data rescues in the biomedical and bio-industrial community.

I have read this submission. I believe that I have an appropriate level of expertise to confirm that it is of an acceptable scientific standard.

# Peer review: Referee response for version 1

**DOI:** 10.5256/f1000research.8516.r12391

**Published:** 2016-02-22

**Authors:** Mark Parsons

**Affiliations:** 1 Research Data Alliance, Troy, NY, USA

This paper advances our understanding of how data are used and referenced. It is well-written, well-referenced, and the methods are appropriate. The data are explained and seem to be available and usable. The conclusions are reasoned and sound. The paper should be approved, and I suggest some minor improvements that would help clarify the methods and make the document more relevant and useful to a broader audience.

The paper analyzes the data citation landscape in the life sciences, specifically around key databases (ENA, PDB) and the open access journals in EPMC and the corpus of patent documents in SureChEMBL. The study findings, however, have implications well beyond life sciences. As such, the paper would benefit from more explanatory context. Some of this explanation was in the discussion section, but it would be helpful to the general reader if more of this was included in the introduction. 

Also I consider myself expert in issues of data citation, but my work has largely been in the geosciences, which has a different data and literature publishing workflow. I was a little confused by the distinction between deposition citations and reuse citations. I think I had it figured out by the end of the paper, but it would have helped to have a clear explanation, early in the article, of the typical biomedical data and article publication process and how that defines a distinction between deposition citations and reuse citations. I was also not fully clear on how deposition and secondary citations were distinguished in the methodology.

Similarly, a bit more detail on the methodology would be useful. For example, the references explain the text-mining techniques, but another paragraph or two in this article that summarized the approach would help the reader understand the approach.

Finally, while I found the discussion and conclusion sections strong, I was thrown by the last sentence in the first paragraph of the discussion. Is this to imply that data citation decreases as a database becomes more established? (I do not have access to the cited article). If that is what is meant, there should be more discussion on the implications for further citation analysis work of this type.

All in all, a useful and important paper. I would be very intrigued to see the methodology applied to other disciplines.

I have read this submission. I believe that I have an appropriate level of expertise to confirm that it is of an acceptable scientific standard.
